# What Prompted the Adoption of Self-Protective Behaviors in Response to COVID-19? Evidence From Women Living in the Rural Areas of Western China

**DOI:** 10.3389/fpubh.2021.756933

**Published:** 2022-01-28

**Authors:** Ruixue Ye, Yuju Wu, Chang Sun, Qingzhi Wang, Yuping Mao, Wei Chang, Huan Zhou

**Affiliations:** ^1^Department of Health Behavior and Social Medicine, West China School of Public Health and West China Fourth Hospital, Chengdu, China; ^2^Department of Communication Studies, College of Liberal Arts, California State University Long Beach, Long Beach, CA, United States; ^3^Harvard Chan School of Public Health, Boston, MA, United States

**Keywords:** perceived efficacy, self-protective behaviors, COVID-19, women, rural western China

## Abstract

**Background:**

Self-protective behaviors, such as handwashing and mask-wearing, are effective to reduce the spread of coronavirus disease (COVID-19), but few studies have focused on women living in rural areas who bear the brunt of the impacts of the pandemic due to their economic and social vulnerabilities. This study explores what prompted the adoption of self-protective behaviors in response to COVID-19 among women living in rural areas of western China.

**Methods:**

The study sample consisted of 1,524 women from 116 townships across 10 counties in rural western China. We collected data in May and August 2020 on women's socioeconomic characteristics, exposure to COVID-19-related information, psychological response to COVID-19, and adoption of self-protective behaviors. Structural equation modeling (SEM) analyses were conducted to analyze the relations among the variables.

**Results:**

During the lockdown, 1,221 (80.12%) of the 1,524 women in the study sample reported wearing a mask every time when they went outside and 1,021 (66.99%) reported handwashing with soap every time after they came home. Perceived efficacy had the strongest association with self-protective behaviors (β = 0.38; *p* < 0.001). Receiving public health guidance (β = 0.18; *p* < 0.001) was indirectly associated with more self-protective behaviors via greater perceived efficacy. Higher socioeconomic status was also directly associated with increased adoption of self-protective behaviors (β = 0.24; *p* < 0.001). Other variables, such as receiving surveillance and risk information, communication channels, perceived risks, and fear, were indirectly associated with the adoption of self-protective behaviors with smaller effect sizes (all β were lower than 0.10).

**Conclusions:**

Not all women were able to adopt self-protective behaviors, such as mask-wearing and handwashing, during the COVID-19 pandemic in western China. To further encourage behavioral changes in response to public health crises, the government should develop clear and actionable guidelines and adopt targeted health communication strategies to reach the most disadvantaged groups of society. These findings may inform tailored responses to COVID-19 in other low- and middle-income countries.

## Introduction

The novel coronavirus disease (COVID-19) has become not only a public health crisis but also a serious threat to social and economic development (1, 2). According to the World Bank, COVID-19 has triggered the deepest global recession in decades, led to a 5.2% contraction in the global economy, and left lasting scars on productivity (1). Within China, COVID-19 was first identified in December 2019 and declared a Chinese national emergency on January 29, 2020. As of August 2021, there were more than 93,100 confirmed cases in China (3). Evidence indicated that the control measures to reduce the spread of COVID-19 resulted in a substantial productivity loss that amounted to over US$382 billion in China (4).

In response to the COVID-19 outbreak, the government worldwide adopted a series of prevention and control measures, including early reporting and situation monitoring, large-scale contact tracing, and health communication campaigns (5, 6). International evidence from USA, Iran, UK, and China showed that people in resource-limited communities are generally more likely to have worse health behaviors during COVID-19 (7–11). In the USA, only 20% of rural participants wore a mask, compared to 47% of urban participants (7). In Iran, 45.7% of participants washed their hands regularly, and handwashing was less common among rural than urban residents (8). Additionally, one UK study indicated that better practice in mask-wearing was significantly associated with living in an urban environment (12). In particular, a study in China indicated that 72.22% of rural residents and 85.70% of urban residents wore a mask when they were outside (10). Compared with urban residents, rural residents are less aware of disease prevention and control measures because of their remote residence and poorer economic conditions (9, 11). In addition, 60% of rural residents are migrant workers in China. Their mobility could have increased rural communities' risk of infection (11). The rural area has become an important battlefield for epidemic prevention and control (13).

Evidence also indicated that gender-responsive policies are required to avoid worsening health and social inequities associated with the COVID-19 pandemic. Specifically, women on average report more days of poor physical and mental health than men despite utilizing more preventive care services (14). These health inequities are larger in women with intersecting identities, such as those living in rural areas (14, 15), potentially due to limited access to healthcare resources and inequitable gender norms that may lead to delayed or forgone healthcare and worse health outcomes (16–18). In addition, as the main caregivers in the family, women often have to spend more time on unpaid domestic work due to the lockdown measures in many countries, including China. Previous studies showed that women often make key dietary choices for their families and act as role models for their children regarding healthy behaviors (19, 20). However, despite their greater burden, lack of support, and potential of influencing other family members, few studies have looked at how to support behavioral changes to promote health and reduce disease risks among women living in rural areas of western China (21–23).

Previous studies underscored the associations between individuals' socioeconomic status, exposure to health information, psychological responses, and behavioral responses during COVID-19. A study in Germany suggests that educational background was positively associated with protective behaviors among the general public (24) while a study from Switzerland suggests the opposite association among young people (25). In Bangladesh, information on the proper use of protective measures from the government were identified as the drivers of COVID-19 protective behaviors (26). Findings from Nigeria and the USA showed that individuals who perceived greater risks of COVID-19 infection were more likely to adopt protective behaviors (27, 28). In China, a study conducted in primary schools showed that educational background of the mother was associated with handwashing and mask-wearing practices of the children (29). Another study in China indicated that perceived risk and severity of the participants were associated with excess protective behavior (30). One study indicated that perceived susceptibility to COVID-19 was associated with consistent mask-wearing among Chinese pregnant women (31). However, no studies have examined the direct and indirect associations among these variables, especially among women in rural areas of western China.

To develop the conceptual framework for our analysis, we turned to the Extended Parallel Process Model (EPPM) that examines how media exposure influences perceptions and behaviors of people in the midst of public health emergencies (32–34). The main components of EPPM include exposure to message communication, perceived efficacy, perceived risk, fear, and behaviors. According to the EPPM, when individuals are exposed to a health message, they would make a cognitive appraisal of the message, including the appraisal of risk and the appraisal of efficacy, which are two key factors that influence the health behavior in response to the health risk. The EPPM also suggests that exposure to health communication messages leads to the belief that individuals are able to reduce the risk (higher perceived efficacy), which in turn prompts individuals to adopt protective behaviors. On the other hand, if individuals do not have enough confidence in their ability to act (lower perceived efficacy), they would not adopt protective behaviors (32–34).

Informed by the evidence from the literature and the EPPM, our study examines the relations among socioeconomic status, exposure to COVID-19-related information, psychological response to COVID-19, and self-protective behaviors among women in rural western China. We hypothesized that there are direct and indirect effects among these variables.

## Materials and Methods

### Study Design, Participants, and Data Collection

The study sample included participants from two surveys. The first survey was part of an ongoing randomized control trial (RCT) among caregivers of children in rural areas of western China. We conducted the baseline survey in May 2020 (35, 36). For this RCT, we used a multistage cluster sampling method to select the study sample. First, we randomly selected four counties from the list of national poverty-stricken counties (defined as counties with an average annual net income of <2,300 RMB, about $1.9 per day) in Sichuan Province (37, 38). Second, 20 townships were randomly selected within each sampled county. Townships that housed the county seat (which are typically more urbanized) were excluded. A total of 80 townships were selected. Third, all primary caregivers with a child aged under 6 months were recruited for the baseline survey. A total of 829 female caregivers were included as the first sub-sample of the present study.

The second survey was from a cohort study among caregivers of the children in rural areas of western China. The cohort study focused on the adherence of the caregivers to a micronutrient home fortification program. We completed the cohort study in August 2020 (39, 40). We used a similar multistage cluster sampling method to select our sample. First, six rural counties were randomly selected from the list of national poverty-stricken counties in Sichuan Province (37, 38). Second, six townships were randomly selected within each sampled county. Townships that housed the county seat were excluded as well. A total of 36 townships were selected. Third, all primary caregivers with a child aged under 24 months were enrolled in the cohort study. In the last round of the data collection for the cohort study, a total of 823 primary caregivers completed all questions on socioeconomic status, health communication, psychological responses, and behavioral responses during COVID-19. Since we focused on women from rural areas in the present study, we excluded 116 male caregivers from the cohort study. Thus, 723 women from the cohort study were included as the second sub-sample of the present study.

Both the 80 townships in the first survey and 36 townships in the second survey were from the list of national poverty-stricken counties in Sichuan Province (37, 38). In total, we included 1,552 participants from the RCT or the cohort study in the present study. Of the 1,552 caregivers who enrolled in this study, 1,494 participants (96.3%) completed the survey. An additional 30 women failed to answer the questions on household assets and health communication, and we imputed these missing values using the regression-based imputation method. Our final analytical sample includes 1,524 participants.

In the first survey, trained enumerators collected data through telephone interviews using a structured questionnaire. In the second survey, trained enumerators used the same questionnaire and collected data via face-to-face interviews. Studies were approved by the Sichuan University Medical Ethical Review Board (approval number of studies: KS2020246). All participants provided written or oral informed consent to participate in the study.

### Measurements

The main variables included demographic and socioeconomic characteristics, exposure to COVID-19-related information, psychological response to COVID-19, and self-protective behaviors in response to COVID-19.

#### Demographic and Socioeconomic Variables

Demographic and socioeconomic characteristics included age, educational level (high school or above), occupation, and household asset level based on household's ownership of or access to a water heater, washing machine, refrigerator, air conditioner, television, computer, motorcycles, and car or truck.

#### Exposure to COVID-19-Related Information

Exposure to COVID-19-related information consisted of communication channels and messaging content. Communication channel was measured by the question “from which channel did you receive most COVID-19 information?” with three response options that included social media (e.g., WeChat, QQ, and TikTok), traditional media (e.g., radio, leaflets, posters, bulletin boards, and newspapers from village and township officials), and interpersonal communication (e.g., face-to-face conversations with family, relatives, and friends). Messaging content was measured by the question “what type of information related to COVID-19 did you received most?” with two response options that included public health guidance (e.g., government-endorsed individual precaution measures, science briefs on disease origin and transmission, infection control measures from the government, and information on local supply of daily necessity and personal protective equipment) and surveillance and risk information (such as risks of infection for family members, local statistics on new cases, the trend of in-migration which might increase a community's risk of exposure to COVID-19, and the epidemic in high-risk areas). All items that measure exposure to COVID-19-related information were binary variables (0 = no, 1 = yes).

#### Psychological Response to COVID-19

We defined psychological response to COVID-19 as a multi-dimensional construct based on empirical evidence (32, 34, 41–44), which included perceived efficacy, perceived risk, and fear emotion. First, according to the EPPM, perceived efficacy was defined as the effectiveness, feasibility, and ease with which a recommended response impedes or averts a threat, such as self-efficacy and response-efficacy (41, 42). Perceived self-efficacy refers to “beliefs about one's ability to perform the recommended response to avert the threat” (41, 42), which was measured with the question “when taking precautions measures, if you had a problem, to what extent do you believe you could address it well.” Perceived response-efficacy is “beliefs about the effectiveness of the recommended response in deterring the threat” (41, 42). Perceived response efficacy was measured by the question “to what extent do you believe that your personal precaution could protect you from getting infected with the coronavirus.”

Perceived risk is the subjective evaluation of the risk contained in the message (43), which is a cognitive construct that comprises two dimensions: perceived susceptibility and perceived severity (34, 43, 44). Perceived susceptibility refers to the perceived likelihood that the risk will directly affect an individual (34, 43, 44), which was measured with two items: “What was the probability of getting infected with COVID-19 to you?” and “What was the probability of getting infected with COVID-19 to your family members?” Perceived severity describes the perceived seriousness of the risk (34, 43, 44), which was assessed by two items: “If you were infected with COVID-19, did you think that it was very fatal for you?” and “If your family members were infected with COVID-19, did you think that it was very fatal for them?”

Fear is conceptualized as a negative emotional reaction to a perceived threat (32, 43). The mood adjectives were the most common measures of self-reported fear in related studies (32). In our study, two items were used to assess fear, i.e., “whether COVID-19 made you feel scared?” and “whether COVID-19 made you feel anxious?”

#### Self-Protective Behaviors in Response to COVID-19

Self-protective behaviors in response to COVID-19, the outcome variables of this study, were defined as behavioral responses of the participants to the COVID-19 pandemic. This definition was based on the guidelines from the WHO for healthy people in response to COVID-19 and previous studies (24, 25, 28, 30, 45). Self-protective behaviors in our study included mask-wearing, measured by the question “during the lockdown, did you wear a mask when you went outside?” and handwashing, measured by the question “during the lockdown, did you wash hands using the soap or detergent when you came back home?” Responses to both questions were rated on a 5-point Likert scale that ranged from 1 for “not at all” to 5 for “very frequent.”

### Statistical Analysis

**Figure 1:**
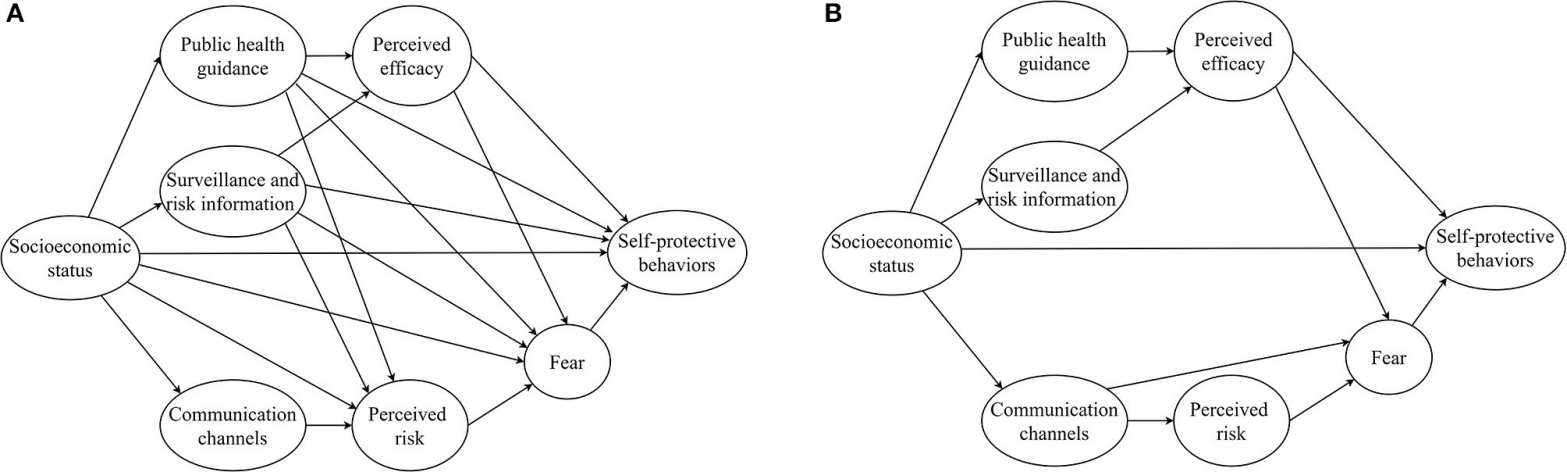
Basic and parsimonious model to map the associations between socio-economic status, exposure to COVID-19-related information, psychological response during COVID-19, and self-protective behaviors in response to COVID-19. **(A)** The basic model. **(B)** The parsimonious model. (Data source: Authors' study).

We first conducted descriptive analysis by computing means and SDs for continuous variables and frequencies and percentages for categorical variables. Based on the EPPM, we hypothesized that women's socioeconomic status, exposure to COVID-19-related information, and psychological response to COVID-19 would have direct and indirect effects on self-protective behaviors. To examine the relations among these variables, we conducted structural equation modeling (SEM) analyses. In this study, SEM was conducted using latent variables (not directly observed but estimated from directly measured variables) and measured variables (directly observed variables). We first included all potential pathways between the variables in a basic model (panel A of Figure 1). Results were presented as standardized β coefficients. The association is considered to be statistically significant if the value of 2-sided *p* is smaller than 0.05. We then fitted a more parsimonious model by removing pathways that had a value of *p* 0.05 or higher (panel B of Figure 1). Multiple tests were used to evaluate the fitness of the model, including normed fit index (NFI), goodness-of-fit index (GFI), comparative fit index (CFI), root mean square error of approximation (RMSEA), and Chi squared/degrees of freedom (χ^2^/*df* ).

Data analyses were performed with Stata statistical software (version 14.1, StataCorp, College Station, TX, USA) and AMOS 21.0 statistical software (IBM, Armonk, NY, USA).

## Results

### Descriptive Statistics of Women in Rural Western China

Table 1 shows the individual characteristics of the study sample. The average age of the sample was 32.7 years (SD = 11.2). Among the 1,524 women in the sample, only 28.5% completed high school education and more than half were stay-at-home parents. Regarding the household economic status, nearly halfowned a lower or low level of household fixed asset (42.5%; panel A of Table 1). Most women had been exposed to COVID-19-related information via social media (52.9%) or traditional media (15.9%), but few (5.3%) had face-to-face conversations with family or friends about this topic. In terms of specific messaging content, <10% of the participants reported having received any COVID-19-related information we asked about in the questionnaire. The only exception is government policies, where 14.4% of the participants reported having received the information of infection control measures from the government (panel B of Table 1).

**Table 1:** Descriptive statistics of women in rural western China (*N* = 1,524).

**Descriptive statistics**	**Mean (std. dev.)/No. (%)**
**Panel A: Demographic and socioeconomic variables**	
Age (year)	32.7 (11.2)
High school or above	434 (28.5)
Stay-at-home parent	977 (64.1)
Household fixed asset lev	
Low	282 (18.5)
Lower	365 (24.0)
Higher	484 (31.7)
High	393 (25.8)
**Panel B: Exposure to COVID-19-related information**	
**Communication channels**	
Social media (e.g.,WeChat, QQ, Kuai Shou, and Dou Yin.	806 (52.9)
Traditional media (e.g., radio, leaflets, posters, bulletin boards, newspapers from village and township officials)	243 (15.9)
Interpersonal communication (e.g., face-to-face conversations with family, relatives, and friends)	81 (5.3)
**Public health guidance**	
Government-endorsed individual precaution measures	52 (3.4)
Science briefs on disease origin and transmission	104 (6.8)
Infection control measures from government	219 (14.4)
Information on local supply of daily necessity and personal protective equipment	114 (7.5)
**Surveillance and risk information**	
Risks of infection for family members	49 (3.2)
Local statistics on new cases	97 (6.4)
Trend of in-migration which might increase a community's risk of exposure to COVID-19	35 (2.3)
The epidemic in high-risk areas	37 (2.4)
**Panel C: Psychological response to COVID-19**	
**Perceived efficacy**	
The confidence one's to solve problems when preventing COVID-19	
Not at all	36 (2.4)
Probably not	86 (5.6)
Neutral	330 (21.7)
Probably	662 (43.4)
Definitely	410 (26.9)
The confidence to protect oneself from getting infected COVID-19	
Not at all	6 (0.4)
Probably not	63 (4.1)
Neutral	273 (17.9)
Probably	794 (52.1)
Definitely	388 (25.5)
**Perceived risk**	
The probability one's infected with COVID-19	
Not at all	595 (39.0)
Probably not	605 (39.7)
Neutral	173 (11.4)
Probably	124 (8.1)
Definitely	27 (1.8)
The probability one's family infected with COVID-19	
Not at all	600 (39.4)
Probably not	603 (39.6)
Neutral	186 (12.2)
Probably	118 (7.7)
Definitely	17 (1.1)
COVID-19 is a serious disease to oneself	
Not at all	57 (3.7)
Probably not	236 (15.5)
Neutral	257 (16.9)
Probably	595 (39.0)
Definitely	379 (24.9)
COVID-19 is a serious disease to one's family	
Not at all	73 (4.8)
Probably not	224 (14.7)
Neutral	275 (18.0)
Probably	622 (40.8)
Definitely	330 (21.7)
**Fear**	
COVID-19 makes oneself scared	
Not at all	221 (14.5)
Probably not	187 (12.3)
Neutral	274 (18.0)
Probably	540 (35.4)
Definitely	302 (19.8)
COVID-19 makes oneself anxious	
Not at all	364 (23.9)
Probably not	243 (15.9)
Neutral	347 (22.8)
Probably	394 (25.9)
Definitely	176 (11.5)
**Panel D: Self-protective behaviors in response to COVID-19**	
Wearing mask when go outside during the lockdown	
Not at all	16 (1.1)
Not often	14 (0.9)
Sometimes	69 (4.5)
Often	204 (13.4)
Very frequent	1221 (80.1)
Washing hands using soap or detergent when came back home during the lockdown	
Not at all	35 (2.3)
Not often	24 (1.6)
Sometimes	133 (8.7)
Often	311 (20.4)
Very frequent	1021 (67.0)

In terms of the psychological response to COVID-19, 70.3% of participants believed that they could cope with the problems related to COVID-19 and 77.6% believed that they could protect themselves from getting infected. A small proportion of participants thought both themselves and their family members were likely to be infected (9.91 and 8.86%, respectively). However, if they were infected, nearly two-thirds of participants believed that COVID-19 would have had severe consequences to themselves (63.91%) and to their family members (62.46%). Over half of the participants reported feeling scared and 37.40% reported feeling anxious about COVID-19 (panel C of Table 1). Regarding self-protective behaviors in response to COVID-19, 80.12% of women wore a mask every time when they went outside and 66.99% of women used soap or detergent to wash hands every time after they came back home (panel D of Table 1).

### Associations Between Latent Variables and Measured Variables

**Figure 2:**
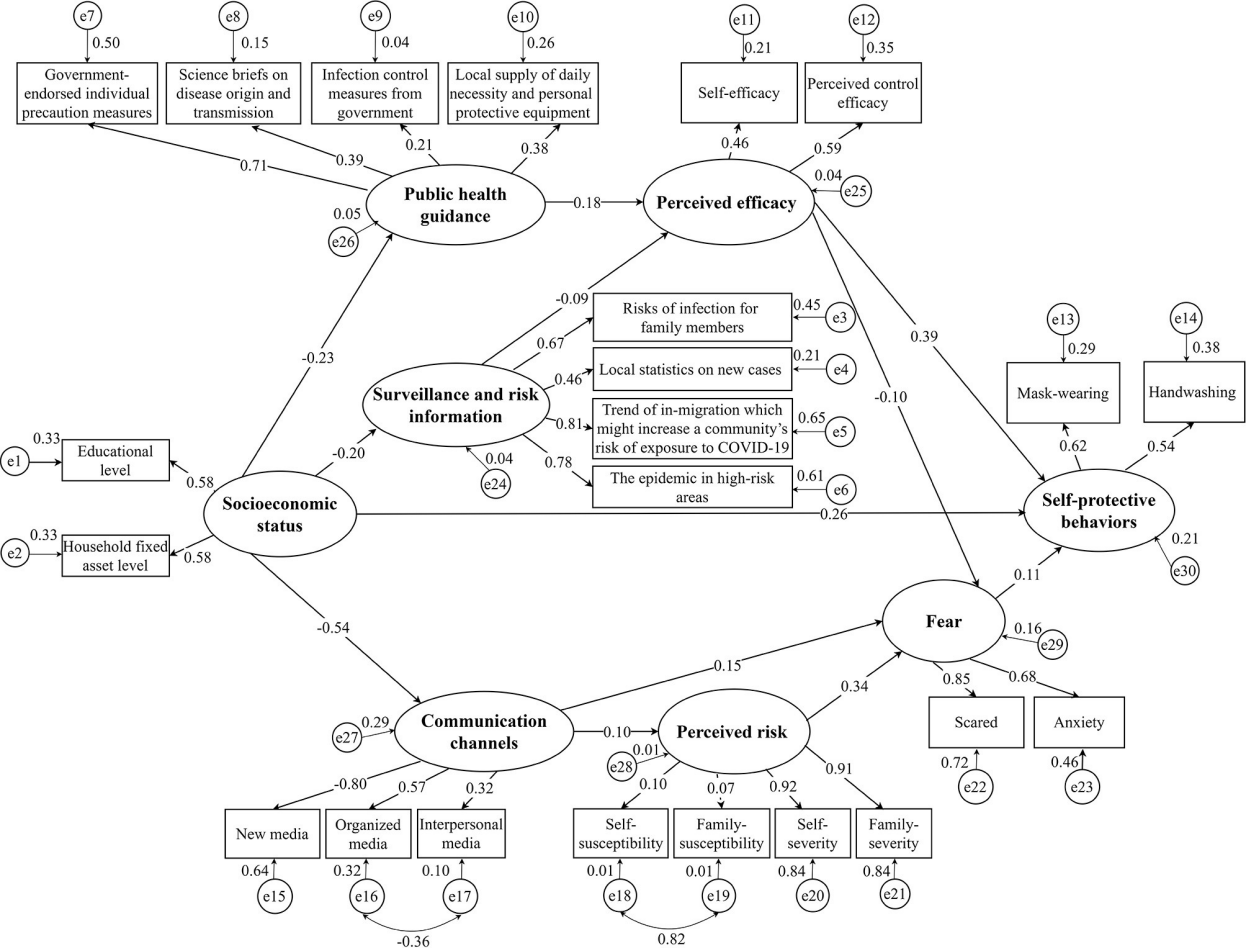
Structural equation modeling examining pathways to self-protective behaviors in response to COVID-19 among women in rural western China (*N* = 1,524). The final adjusted model had a better fit than the basic model with the following indicators: NFI = 0.85, GFI = 0.94, CFI = 0.87, RMSEA = 0.06, and χ^2^/df = 5.88. Coefficients are standardized path coefficients. Variables in ellipse represent latent variables, in squares represent observed variables. Self-efficacy indicates the confidence one's to solve problems when preventing COVID-19. Perceived control efficacy indicates the confidence to protect oneself from getting infected COVID-19. Self-susceptibility indicates the probability of one's being infected with COVID-19. Family-susceptibility indicates the probability of one's family being infected with COVID-19. Self-severity indicates COVID-19 is a serious disease to oneself. Family severity indicates COVID-19 is a serious disease to one's family. Scared indicates COVID-19 makes oneself scared. Anxiety indicates COVID-19 makes oneself anxious. NFI, normed fit index; GFI, goodness-of-fit index; CFI, comparative fit index; RMSEA, root mean square error of approximation. (Data source: Authors' study).

Associations between latent variables and measured variables are shown in Figure 2. The measured variables were adequate indicators of respective latent variables. For example, participants' educational level (β = 0.58, *p* < 0.001) and household fixed asset level (β = 0.58, *p* < 0.001) were adequate indicators of social economic status (SES). Self-efficacy (β = 0.46, *p* < 0.001) and perceived control efficacy (β = 0.59, *p* < 0.001) when encountered the difficulties of preventing COVID-19 were used to represent the perceived efficacy variables. Self-protective behaviors were well-represented by mask-wearing (β = 0.62, *p* < 0.001) and handwashing (β = 0.54, *p* < 0.001).

### Factors Associated With Self-Protective Behaviors in Response to COVID-19

Results from the SEM analyses are presented in Table 2. Perceived efficacy had the strongest association to self-protective behaviors with the standardized total effect of 0.38, including a direct effect of 0.39 and an indirect effect of −0.01 through fear. We further examined the factors associated with perceived efficacy. Table 2 indicates that receiving public health guidance had the strongest correlation with perceived efficacy, the standardized total effect is 0.18.

**Table 2:** Standardized direct, indirect, and total effects of dominants on self-protective behaviors and perceived efficacy in response to COVID-19 among women in rural western China (*N* = 1, 524).

**Variables**	**Total effect**		**Direct effect**		**Indirect effect**	
	**Self-protective behaviors**	**Perceived efficacy**	**Self-protective behaviors**	**Perceived efficacy**	**Self-protective behaviors**	**Perceived efficacy**
Socioeconomic status	0.24	–	0.26	–	−0.02	−0.02
Communication channels	0.02	–	–	–	0.02	-
Public health guidance	0.07	0.18	–	0.18	0.07	-
Surveillance and risk information	−0.04	−0.09	–	−0.09	−0.04	-
Perceived efficacy	0.38	–	0.39	–	−0.01	-
Perceived risk	0.04	–	–	–	0.04	-
Fear	0.01	–	0.11	–	–	-

Social economic status was also directly and indirectly correlated with self-protective behaviors. The standardized total effect of SES was 0.24, with a direct effect of 0.26 and an indirect effect of −0.02, mainly through public health guidance, surveillance and risk information, or communication channels. Other perceived risks and fear were indirectly correlated with self-protective behaviors, but the effects were smaller with a coefficient <0.10.

In Table 3, each latent variable in the model is further broken down into its components to assess the relations of these measured variables with self-protective behaviors. Among the measured variables, perceived control efficacy was the most powerful predictor with a correlation coefficient of 0.22. Within the SES indicators, the educational level and household fixed asset level had the same correlation coefficient of 0.16 with self-protective behaviors. As to perceived efficacy, government-endorsed individual precaution measures were the strongest predictors of self-protective behaviors, the correlation coefficient was 0.13.

**Table 3:** Relationship of the measured variables, latent variables with self-protective behaviors and perceived efficacy in response to COVID-19 among women in rural western China (*N* = 1524).

**Variables**	**Standardized regression coefficients**	
	**Self-protective behaviors**	**Perceived efficacy**
**Socioeconomic status**	**0.24**	–
Educational level	0.14	–
Household fixed asset level	0.14	–
**Communication channels**	**0.02**	–
Social media (e.g., WeChat, QQ, Kuai Shou, and Dou Yin.	0.02	–
Traditional media (e.g., radio, leaflets, posters, bulletin boards, newspapers from village and township officials)	0.01	–
Interpersonal communication (e.g., face-to-face conversations with family, relatives, and friends)	0.01	–
**Public health guidance**	**0.07**	**0.18**
Government-endorsed individual precaution measures	0.05	0.13
Science briefs on disease origin and transmission	0.03	0.07
Infection control measures from government	0.01	0.04
Information on local supply of daily necessity and personal protective equipment	0.03	0.07
**Surveillance and risk information**	**0.04**	**0.09**
Risks of infection for family members	0.03	0.06
Local statistics on new cases	0.02	0.04
Trend of in-migration which might increase a community's risk of exposure to COVID-19	0.03	0.07
The epidemic in high-risk areas	0.03	0.07
**Perceived efficacy**	**0.37**	–
The confidence one's to solve problems when preventing COVID-19	0.17	–
The confidence to protect oneself from getting infected COVID-19	0.22	–
**Perceived risk**	**0.04**	–
The probability one's infected with COVID-19	0.00	–
The probability one's family infected with COVID-19	0.00	–
COVID-19 is a serious disease to oneself	0.04	–
**Fear**	**0.11**	–
COVID-19 makes oneself scared	0.09	–
COVID-19 makes oneself anxiety	0.07	–

*The bold values all highlight standardized regression coefficients of latent variables*.

## Discussion

Women in rural areas of China are disproportionally affected by COVID-19 but little is known about how they cope with the pandemic and whether they could adopt self-protective behaviors to reduce their risks of infection. This study reached a relatively neglected and vulnerable sample during the peak period of the COVID-19 outbreak, which found that not all women were able to adopt self-protective behaviors, such as mask-wearing and handwashing during the COVID-19 pandemic in western China. By examining the associations between exposure of individuals to COVID-19-related information, psychological response to COVID-19, and self-protective behaviors, we found that perceived efficacy and socioeconomic backgrounds were important factors directly associated with the adoption of self-protective behaviors during the COVID-19 pandemic among women in rural western China. Moreover, exposure to information of public health guidance was associated with self-protective behaviors indirectly via increased perceived efficacy. Such findings may help in tailoring efficacious interventions for improving the COVID-19 pandemic response among women in rural western China.

In the ongoing COVID-19 pandemic, non-pharmaceutical protective measures, such as mask-wearing and handwashing, remain crucial. In our study, most women were able to adopt these protective behaviors (80.1% in mask-wearing and 60.0% in handwashing), which is higher compared to another study in Nigeria where 37.7% of women from the rural areas reported always wearing a face mask when going out and 46.8% responded always washing hands (46), also higher than another study in African where 22.5% of women from the rural areas reported good practice of preventive measures against COVID-19 infection, including hand washing, wearing a mask, and maintain social distance (47). But our behavior results were lower than those reported in an Ecuadorian study where the local women had a higher engagement in mask-wearing (91.9%) and handwashing (96.7%) (48) and still lower than an Iranian study where the local women had a high performance in protective behaviors (97.3%) (49). We additionally found a different trend between the behavior results from ours and the international study. Our study found that mask-wearing was more common than handwashing, which differs from the findings of the above Nigeria and Ecuadorians studies (46, 48), also differs from the studies in Ethiopia and the USA. A study in Ethiopia found that half of the women (51.61%) would wear masks compared with that 90% of wash hands (50). Similarly, in the USA, handwashing (87.2%) was common than mask-wearing (23.1%) among women (51). This difference could be explained by the fact that the public in China is more open to mask-wearing due to the experience of previous outbreaks, such as the severe acute respiratory syndrome (52), early recognition of asymptomatic transmission, government's effort to increase public awareness of the importance of mask-wearing, and strict enforcement of the mask mandate (53).

We found that greater perceived efficacy in coping with COVID-19 is associated with increased self-protective behaviors, which echoes the findings from a growing number of studies that demonstrate the link between self-protective behavior and efficacy (31, 34, 54, 55). EPPM proposed that when faced with health risks, people would perceive that they themselves are able to perform recommended self-protective behaviors and that these behaviors are effective in responding to the threat. This perception would in turn lead to more self-protective behaviors. On the other hand, this result offers new insight into strategies to promote health behaviors through enhancing response efficacy. The reason may be that response efficacy was a type of action perspective to remove the risk (54, 56), individuals are more likely to adopt self-protective behaviors if they believe they can easily, feasibly, and effectively prevent a health threat with serious consequences. Greater perceived response efficacy may indicate stronger confidence that guideline-recommended measures are effective in reducing the risk of COVID-19 infection, which in turn would increase individuals' protective behaviors. More research is needed to identify effective strategies to increase perceived efficacy, especially response efficacy, to promote self-protective behaviors against COVID-19 and other infectious disease crises.

Furthermore, according to EPPM, the behavioral response is directly affected by perceived efficacy of people, which can be targeted by health messages (54, 56). We examined this potential association and found that exposure to public health guidance, particularly the government-endorsed individual precaution measures, was associated with greater perceived efficacy. Previous evidence has shown that official reports by the government and health education campaigns that promote self-protective behaviors were critical in slowing the spread of disease (57, 58). Most women in rural areas are not well-educated and have limited household assets (59, 60). Thus, it might be difficult for them to access health information from a variety of sources (39). To them, the public health guidance from the government could be the most reliable and accessible information source during the COVID-19 pandemic (61). In addition, clear and specific guidance on individual protective measures, such as how to properly and effectively wear masks and wash hands, may have enhanced individuals' confidence in engaging in self-protective behaviors. To reach a broad audience, the government should use specific and actionable messages in their health communication campaigns against COVID-19.

Although communication channel, risk perception, and fear were direct or indirect predictors of behavioral outcomes, they only had modest effects on self-protective behaviors in this study. As explained by EPPM (32, 33), response of an individual to a risk-based message involves two distinct cognitive appraisals. The first appraisal is related to the degree to which the message is perceived as threatening, i.e., perceived susceptibility and severity. If the individual perceives that they are personally vulnerable and that the risk is severe, the second appraisal occurs whereby the individual considers whether the message provides effective and useful strategies to reduce the risk. In other words, perceived susceptibility and severity alone are not sufficient and appraisal of effective actions is needed to prompt actions, as suggested by our primary finding that perceived efficacy was the main precursor of self-protective behaviors.

Consistent with previous studies conducted in China, Brazil, and the United States (62–64), women with higher SES, such as educational and economic levels, were more likely to adopt self-protective behaviors in our study. This might be because women with higher levels of SES had better access to public health information and understand better the government-recommended precautious measures (61, 62). To better promote self-protective behaviors against COVID-19 in the rural areas, health communication interventions should be tailored to accommodate the needs of individuals with low health literacy to reach women from disadvantaged backgrounds.

Taken together, the findings will shed light on direct and indirect factors contributing to the self-protective behaviors during the COVID-19 pandemic among women living rural areas of western China based on EPPM. Within a risk situation, participants may perceive high levels of risk, they may need more information to enhance their efficacy of responding to the risk. The communication message provided therefore should aim at enhancing the efficacy of women from the rural areas by strengthening the government-endorsed individual precaution measures and giving clear guidelines on how the self-protective behaviors can be undertaken, which might be increased their engagement in the self-protective behaviors. SES was also the important precursor of self-protective behaviors, those women with a lower level of SES should be paid more attention during the COVID-19 pandemic. These findings implied to us, that efforts to foster the high efficacious message and strengthen the disadvantaged populations during and in the aftermath of COVID-19 may mitigate the inequitable risks posed by pandemics and other times of healthcare stress.”

## Study Limitations

The findings should be interpreted in the context of a few limitations. First, data in the study were collected after the lockdown was over, we did not collect any data at the beginning of COVID-19, and we were unable to rule out the possibility of recall bias. However, we emphasized in our survey questions that we were asking about experience of the participants “during the lockdown” and trained enumerators to follow a standard survey manual when asking these questions. Second, we collected data only via structured interviews to measure how dynamic individual characteristics, such as exposure to health information and psychological response, affect health behaviors. Future research could incorporate qualitative approaches to develop a more in-depth understanding of the interplay between these factors and the way they affect health behaviors. Third, the analysis sample was constructed from two surveys conducted 3 months apart at the peak of COVID-19 in our study. Despite similarities between these two sub-samples in background characteristics of the participants, the time difference might have introduced biases from unmeasured co-founders. We attempted to analyze the two sub-samples separately, but the model was under-fitting. To get a larger sample size for model fitting, we thus combined the two sub-samples in the analysis. Fourth, our study sample consists of women from rural areas of Sichuan Province, which is the province with the largest population in western China. However, our finding might not be generalizable to other areas of western China due to differences in population characteristics, cultural customs, social norms, and physical environments across various regions of western China (65, 66). Since the majority of previous studies in China were conducted in urban areas (6, 67, 68), future research on women from other resource-limited settings is needed to understand their health behaviors and develop more targeted health communication strategies. Fifth, for women from rural areas with limited resources, access to personal protective materials, such as masks and soap, maybe other environmental determinants of self-protective behavior. A study in China has shown that an adequate supply of masks and soap was associated with higher odds of mask-wearing and handwashing among the general population (68). Unfortunately, we did not measure access or supply in our study.

## Conclusion

This study examined how individual characteristics, exposure to COVID-19-related information, and psychological response to COVID-19 affected self-protective behaviors of women in rural areas of western China. To the best of our knowledge, this is the first study that focused on this often-overlooked vulnerable group and their health behaviors during the peak of the COVID-19 outbreak. Our findings suggested that targeted messages and group-specific risk communication strategies may encourage self-protective behaviors among women from the rural areas of western China. More broadly, to promote behavioral changes in response to public health crises, the government should develop clear and actionable guidelines and adopt targeted health communication strategies to reach the most disadvantaged groups of the society.

## Data Availability Statement

The datasets analyzed for the current study are not publicly available due to ethical restrictions related to the consent given by participants at the time of study commencement. An ethically compliant dataset may be made available by the corresponding author on reasonable request and upon approval by the Sichuan University Medical Ethical Review Board. Requests to access the datasets should be directed to zhouhuan@scu.edu.cn.

## Ethics Statement

The studies involving human participants were reviewed and approved by Sichuan University Medical Ethical Review Board (approval number of studies: KS2020246). The participants provided their written or oral informed consent to participate in this study.

## Author Contributions

RY and YW drafted the paper plan, developed the data analysis plan, and led the writing of the manuscript. RY, YW, CS, QW, and HZ designed the study. YM, WC, and HZ critically reviewed, discussed, and modified the manuscript. All authors contributed to the article and approved the submitted version.

## Funding

This study was funded by Stanford University Research Foundation (Grant Number: 0040405502159) and National Natural Science Foundation of China (Grant Number: 71874114).

## Conflict of Interest

The authors declare that the research was conducted in the absence of any commercial or financial relationships that could be construed as a potential conflict of interest.

## Publisher's Note

All claims expressed in this article are solely those of the authors and do not necessarily represent those of their affiliated organizations, or those of the publisher, the editors and the reviewers. Any product that may be evaluated in this article, or claim that may be made by its manufacturer, is not guaranteed or endorsed by the publisher.
