# Supplementary material for: What Prompted the Adoption of Self-Protective Behaviors in Response to COVID-19? Evidence From Women Living in the Rural Areas of Western China
Source: Front Public Health. 2022 Jan 28;9:756933. doi: 10.3389/fpubh.2021.756933 (PMC8831835; doi:10.3389/fpubh.2021.756933)
Supplement: Supplementary file 1 [file Table_1.DOCX]

Supplementary Material

**The sample size calculation**

The sample size in our study was mainly determined by the study design and the analytical method.

Study design:

Since the two surveys were from different studies, we will describe the details of sample size calculation separately. For the first survey, which is part of the township-based cluster RCT, we determined the study’s sample size by power calculations performed before enrollment. The power to detect a difference in anemia rates between the treatment and control groups in a township-based cluster RCT depends on the following six factors:

(1) numbers of infants per township;

(2) number of townships in the treatment group;

(3) number of townships in the control group;

(4) the intracluster correlation of anemia prevalence;

(5) the hemoglobin level in baseline; and

(6) the treatment effect that we would expect to be able to detect.

We assumed an intracluster correlation of 0.01 and 10 infants per township. Based on these parameters, we calculated that we would require at least 80 townships (40 townships per group) to detect a treatment effect of 0.2 at 80% power given a significance level of .05. Therefore, a total of 800 participants are needed for the RCT. In the present study, we only included female participants; a total of 817 female caregivers completed all questions on socioeconomic status, health communication, psychological responses, and behavioral responses during COVID-19 who were included in the analysis.

For the second survey, which is part of a cohort study, we determined the study’s sample size using the sample size calculation methods of multi-stage random sampling. Specifically, we first used the simple random sampling equation to calculate the sample size (equation 1 as follows). Second, we used the design effect (*Deff*) to adjust the sample size (equation 2 as follows).

$n=\left( \frac{Z_{\propto/2}}{\delta} \right). p\left( 1-p \right)$ （1）

$n_{c}=n. Deff$ （2）

Based on previous studies (36-38), adherence to the home fortification program was 40-80%, which varied in different areas. According to equation (1)，$p$ = 50% was used to estimate the maximum sample size. Using $\propto$= 0.04, α =0.05, $Z_{\propto/2}$= 1.96，the sample size ($n$) was 600. According to equation (2)，$Deff$ = 2 was used to adjust the sample size ($n_{c}$). The adjusted sample size ($n_{c}$) was 1200. Considering the possibilities attrition, we increased the sample size by 15%. Thus, a total of 1320 caregivers are needed in the first round of data collection. In the present study, we included only responses from female participants; a total of 707 female caregivers completed all questions on socioeconomic status, health communication, psychological responses, and behavioral responses during COVID-19 and were included in the analysis.

Analytical method:

The present study combined two surveys that used Structural Equation Model (SEM) to analyze the data. We used the following equation to calculate the sample size for SEM,

$$N_{RMSEA}=\frac{\lambda}{{(\mathrm{RMSEA})}^{2}df}+1$$

According to the above equation, the power of non-central parameter statistical test was set at 0.8, $\lambda$ was 45.81, the equation fitting index ($\mathrm{RMSEA}$) was 0.05. Additionally, the degree of freedom ($df$) was equal to the difference between the number of data points and the number of free parameters of the model in the simulation model. The final sample size is around 500. Therefore, the sample size of the present study would meet the analysis requirements.
